# The antiangiogenic role of the pro-inflammatory cytokine interleukin-31

**DOI:** 10.18632/oncotarget.14857

**Published:** 2017-01-27

**Authors:** Shiri Davidi, Ella Fremder, Tal Kan, Ziv Raviv, Michael Timaner, Nathan Karin, Dov Hershkovitz, Ami Arohneim, Yuval Shaked

**Affiliations:** ^1^ Cell Biology and Cancer Science, Rappaport Faculty of Medicine, Technion, Haifa, Israel; ^2^ Department of Immunology, Rappaport Faculty of Medicine, Technion, Haifa, Israel; ^3^ Department of Pathology, Rambam Medical Center, Haifa, Israel

**Keywords:** metastasis, angiogenesis, cancer therapy, host response

## Abstract

Pro-inflammatory cytokines in the tumor microenvironment are known for their ability to either inhibit or promote cancer progression. Here we evaluated the role of Interleukin-31 (IL31), a protein belonging to the pro-inflammatory IL-6 cytokine family which has been characterized in autoimmune disease, in tumorigenesis. We show that IL31 and its receptor, IL31RA, are highly expressed in various human and mouse cancer cell lines, as well as in tumor specimens from cancer patients. MC38 murine colon carcinoma cells depleted of IL31 exhibit an increase in invasive and migratory properties *in vitro*, effects that are reversed by supplementing the cells with exogenous IL31. *In vivo*, IL31-depleted MC38 tumor cells implanted to mice grow faster than control tumors. In contrast, MC38 tumor-bearing mice infused with recombinant IL31, exhibit a significant reduction in tumor growth than control mice. Furthermore, IL31 infusion reduces the number of metastatic lesions in the lungs of mice bearing 4T1 murine metastatic breast carcinoma. Lastly, injecting tumor-bearing, chemotherapy-treated mice with a long-lived IL31-IgG fusion protein reduces tumor growth, angiogenesis and pulmonary metastasis to a greater extent than when chemotherapy is used alone. The IL31 anti-tumor activity is explained, in part, by the anti-angiogenic effects demonstrated both *in vitro* and *in vivo* highlighting the potential use of IL31 as an anti-cancer drug.

## INTRODUCTION

One of the main obstacles in clinical oncology is the development of resistance to therapy leading to tumor re-growth and even spread. There are several mechanisms underlying tumor resistance. For example, selection of tumor cells bearing genomic mutations leads to acquired resistance. In addition, accessory cells within the tumor microenvironment can protect cancer cells from the drug cytotoxic effects [1–3]. We and others have demonstrated that several types of bone marrow derived cells (BMDCs) including endothelial progenitor cells or Tie-2 expressing monocytes home to chemotherapy-treated tumors, leading to increased angiogenesis and subsequent tumor regrowth [4–6]. Blocking the colonization or function of these host cells using antiangiogenic drugs improves therapy outcome, and delays recurrence [6–8]. These host cellular effects are accompanied by a substantial upregulation of growth factors, enzymes, chemokines and cytokines [7, 9, 10]. For example, it has been demonstrated that following paclitaxel (PTX), 5FU and gemcitabine chemotherapies, IL1-β, an upstream cytokine known to initiate the pro-inflammatory cascade, is highly expressed in various organs. IL1-β activates immune cells such as macrophages at the treated tumor site, therefore supporting tumor regrowth and metastasis [11, 12]. Furthermore, we also found that VEGF-C, a molecule promoting lymphangiogenesis in tumors, is substantially upregulated in the plasma and organs of mice treated with PTX chemotherapy [13]. Specifically, naïve mice implanted with cultured tumor cells together with macrophages obtained from PTX-treated mice exhibited increased tumor lymphangiogenesis, in a similar manner to tumor-bearing mice treated with PTX [13]. These collective studies demonstrate that the host response to chemotherapy results in a substantial increase in various BMDCs and factors that affect the tumor microenvironment and negate the drug anti-tumor activity [1].

We therefore undertook a proteomic approach to screen for additional cytokines and growth factors that are induced in the plasma of mice after chemotherapy, with the aim of identifying those that affect the tumor microenvironment. In this screen, non-tumor bearing mice were treated with various chemotherapy drugs. We found that Interleukin-31 (IL31), a protein belonging to the pro-inflammatory IL-6 cytokine family, was upregulated in the plasma and bone marrow cells following PTX and FOLFOX chemotherapy, respectively (Supplementary Figure 1). IL31 is an immunoregulatory cytokine that is mainly produced by activated T helper (CD4) and cytotoxic T (CD8) cells, and is elevated in atopic dermatitis [14, 15]. IL31 acts through the heterodimer receptors of IL31 (IL31RA) and oncostatin M receptor (OSMR) that are expressed on activated monocytes and unstimulated epithelial cells [16]. A recent study demonstrated that IL31 is highly effective in suppressing the proliferation of lung epithelial cells by upregulating several signaling pathways [18]. The blockade of IL31 has been shown to reduce dermatitis in mice and human [14, 18].

There is only limited evidence for the involvement of IL31 in cancer. Clinically, it has been shown that IL31 correlates with cutaneous T-cell lymphoma progression [19]. Furthermore, a recent study indicated that IL31 decreased the proliferation of HCT116 colorectal cancer cells, a process dependent on cultured cell density [20]. However, the contribution of IL31 to tumor progression or metastasis is still elusive.

In this study we investigated the role of IL31 in cancer progression focusing on processes other than immunomodulation. We found that IL31 and IL31RA are expressed by several types of tumor cells from both mouse and human origins. Mice infused with IL31 exhibited tumor growth retardation and reduced number of pulmonary metastatic lesions. These effects are attributed at least in part to the direct anti-angiogenic role of IL31. Overall, our results demonstrate the potential use of IL31 as a novel antiangiogenic drug for the treatment of cancer.

## RESULTS

### Expression of IL31 and/or IL31RA in cancer cell lines and human tumor specimens

**Figure 1:**
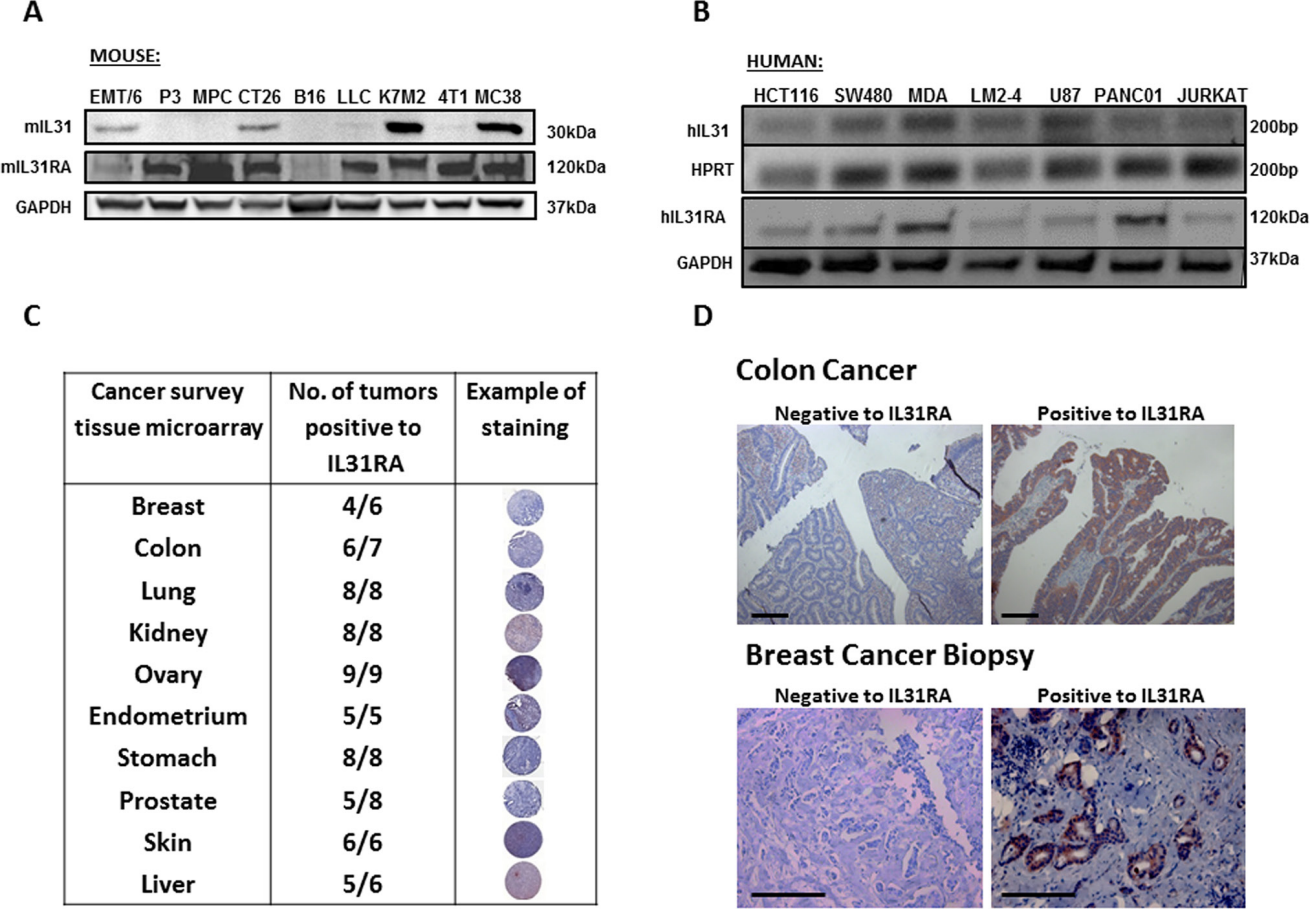
IL31 is expressed in different cancer cell lines and human cancer tissue (**A**) Protein levels of IL31 and IL31RA in the indicated murine cell lines were evaluated by Western blot. (**B**) mRNA levels of IL31 and IL31RA in the indicated human cell lines were evaluated by semi-quantitative PCR (upper panel) and Western blot (lower panel). (**C**) Cancer survey tissue microarray was used to stain IL31RA (brown) and counterstained with hematoxylin. The table demonstrates the expression levels of IL31RA in different malignant tissues. (**D**) Human colon and breast carcinoma specimens were immunostained for IL31RA (brown) and counterstained with hematoxylin. Representative images are provided. Scale bar = 200 μm.

The expression pattern of IL31 and its receptors in various normal tissues from both murine and human origins has been described previously [14]. Here, we investigated the expression of IL31 and its receptor (IL31RA) in various cancer cell lines and human tumor specimens. The mRNA and protein levels of IL31 and IL31RA were evaluated in cell lysates derived from myeloma, leukemia, melanoma, glioblastoma, and several carcinoma cell lines of both mouse and human origins. Of note, the reason for using cell lysates and/or mRNA levels and not conditioned medium extracted from such cells is due to the lack of sensitive antibodies for the detection of IL31 by Western blot, especially for human IL31. The results in Figure 1A–1B demonstrate that the different cell lines express IL31 and IL31RA to various degrees. Specifically, high expression of IL31 was found in 4/9 murine and 5/7 human cell lines. Tissue microarray analysis of human tumors revealed that a high proportion of tumor specimens express IL31RA in all the tested tumor types (Figure 1C). High expression of IL31RA was also found in 10/15 and 12/15 breast and colon carcinoma specimens, respectively (Figure 1D). It should be noted that the majority of tumor cells express the Oncostatin M Receptor, as indicated by the online human protein atlas [21]. Overall these results indicate that certain tumor types highly express IL31 and/or IL31RA, and thus suggest a potential role for the IL31-IL31RA axis in cancer.

### IL31 inhibits cell migration and invasion

**Figure 2:**
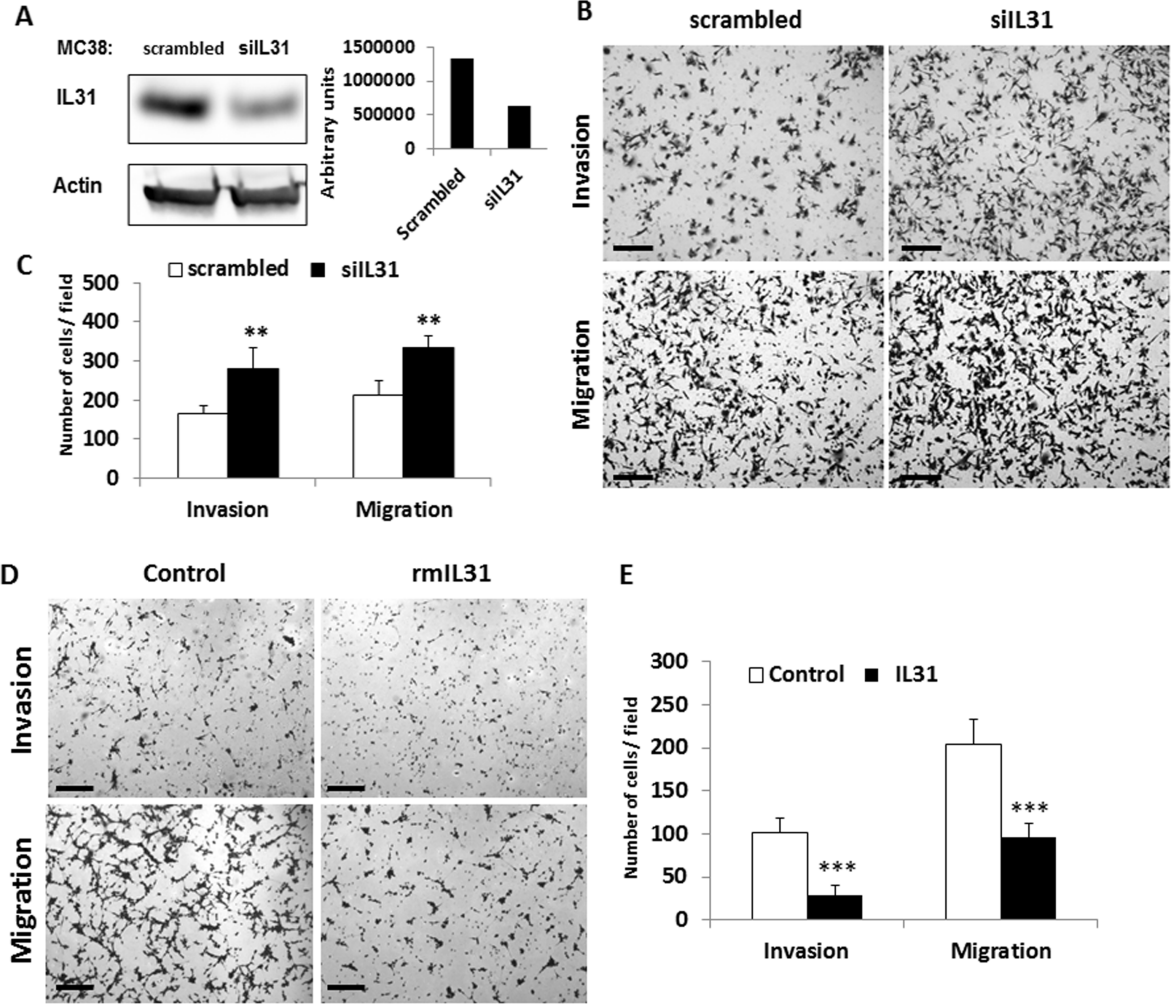
IL31 inhibits tumor cell invasion and migration (**A**) MC38 murine colon carcinoma cells were stably transfected with a control scrambled or siRNA plasmid against IL31. Left panel: Expression of IL31 was assessed by Western blot analysis using anti-IL31 antibodies. Actin served as the loading control. Right panel: IL31 protein levels were quantified by densitometry analysis. (**B**, **C**) The invasion and migration properties of control and siIL31 MC38 cells were evaluated using the Boyden chamber assay. Representative images are shown in (B). Scale bar = 200 μm. Quantification of invading and migrating cells is shown in (C). (**D**, **E**) The invasion and migration properties of control and IL31-treated MC38 cells were evaluated using the Boyden chamber assay. Representative images are shown in (D). Scale bar = 200 μm. Quantification of invading and migrating cells is shown in (E). **, 0.01 > *p* > 0.001; ***p < 0.001.

To investigate the possible role of IL31 in tumor cells, we silenced IL31 expression in MC38 murine colon carcinoma cells that highly express IL31 (Figure 1A) using siRNA (siIL31). A selected siIL31 MC38 clone showed a 50% decrease in IL31 expression levels when compared to control cells stably-transfected with scrambled siRNA (Figure 2A). Next, control and siIL31 MC38 cells were assessed for cell invasion and migration properties. The siIL31 MC38 cells exhibited a significant increase in cell invasion and migration properties in comparison to control (Figure 2B–2C). Consistently, culturing wt-MC38 cells in the presence of IL31 (100ng/ml) reduced invasion and migration properties in comparison to untreated cells (Figure 2D–2E).

### IL31 does not affect tumor cell viability, proliferation, cell cycle, and apoptosis

IL31RA activation involves the MAPK and PI3K signaling pathways and STAT3/5 transcription factors [17, 22]. These signaling cascades contribute to several cell processes including cell proliferation, apoptosis, differentiation and inflammation [23]. Since IL31RA is expressed by several tumor types, we studied whether its activation by IL31 affects the above-mentioned cellular processes. We chose to work with MC38 and CT-26 colon carcinoma cells that highly express both IL31 and IL31RA (Figure 1A). The cells were cultured in the presence of escalating doses of rmIL31, and were evaluated for cell viability by the AlamarBlue assay, proliferation by BrdU assay, as well as cell cycle and apoptosis by flow cytometry as described in Materials and Methods. There were no significant differences in cell viability, proliferation, cell cycle and apoptosis at any of the rmIL31 concentrations tested, up to a dose of 100 ng/ml (Supplementary Figure 2 and data not shown).

### IL31 inhibits tumor growth *in vivo* in part by an anti-angiogenic effect

**Figure 3:**
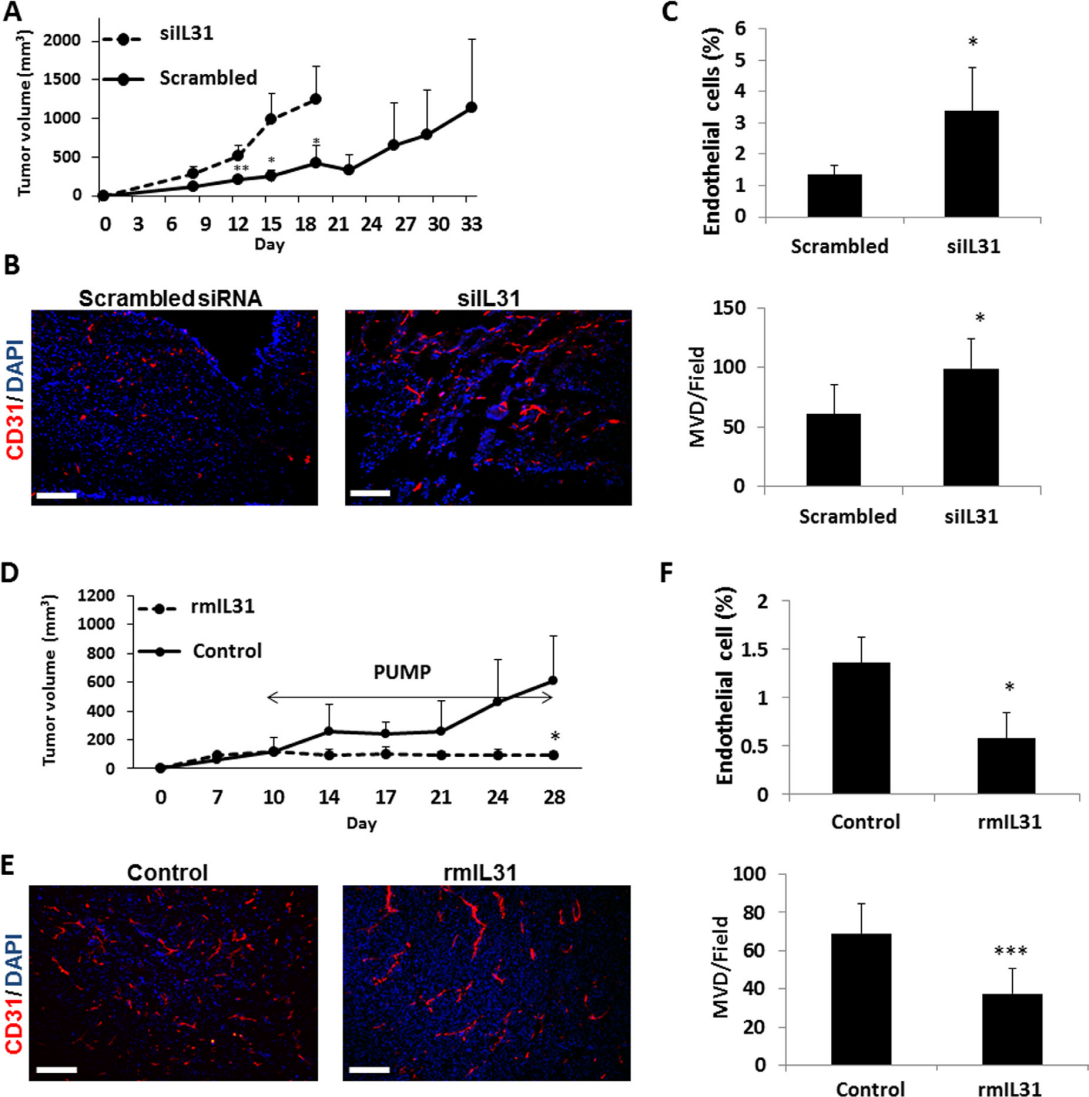
IL31 inhibits tumor growth and angiogenesis (**A**–**C**) Control or IL31 siRNA MC38 cells (1 × 10^6^) were subcutaneously implanted into the flanks of 8-week old C57Bl/6 mice (*n* = 4–5 mice/group). Tumor growth was assessed with a caliper using the formula width^2^ × length × 0.5 (A). At end point, tumors were removed and divided into two equal parts. One part was sectioned (B) and the other part was prepared as a single cell suspension (C). Tumor sections were immunostained for CD31, an endothelial cell marker (red). Nuclei were stained with DAPI (blue). Scale bar = 200 μm (B). Tumor single cell suspensions were assessed for the percentage of endothelial cells by flow cytometry (C). (**D**–**F**) MC38 cells (1 × 10^6^) were implanted into the flanks of 8-week old C57Bl/6 mice (*n* = 4–5 mice/group). Tumors were allowed to grow until they reached a size of 100 mm^3^, at which point mice were implanted with micro-osmotic pumps containing rmIL31 (administered at a dose of 0.7 μg/day) or PBS. Tumor growth was assessed regularly (D). At end point, tumors were removed and divided into two equal parts. One part was sectioned for endothelial cell staining (E) and the other part was prepared as a single cell suspension for the evaluation of endothelial cell percentage by flow cytometry (F), as in (B–C). **, 0.01 > *p* > 0.001; ***p < 0.001.

To assess the effect of IL31 on tumor growth *in vivo*, control or siIL31 MC38 cells were implanted into C57Bl/6 mice and tumor growth was monitored regularly. Tumors from mice implanted with MC38 siIL31 cells grew significantly faster than control tumors (using scrambled siRNA). (Figure 3A). At end point, tumors were harvested, and cryosections were analyzed for microvessel density (MVD) using CD31 as an endothelial cell marker. In addition, harvested tumors were also prepared as single cell suspensions for the evaluation of endothelial cell percentage by flow cytometry. The number of microvessels and the percentage of endothelial cells were significantly higher in siIL31 MC38 tumors in comparison to control tumors (Figure 3B–3C).

Next, we assessed the changes in tumor growth in mice administered with recombinant murine IL31 (rmIL31). Since IL31 is a small cytokine of ∼24 kDa in size, we used subcutaneous micro-osmotic pumps to infuse rmIL31 protein at a dose of 0.7 μg/day for 2 weeks. A complete inhibition of tumor growth was observed in mice infused with rmIL31 compared to control mice infused with vehicle (Figure 3D). The number of microvessels and the percentage of endothelial cells were decreased in tumors harvested from the IL31-treated mice, as assessed by CD31 staining and flow cytometry, respectively. Notably, a larger vessel phenotype was observed in tumors from IL31-treated mice (Figure 3E–3F). Collectively, these results suggest that continuous infusion of IL31 inhibits tumor growth, partly by an anti-angiogenic activity.

### IL31 inhibits metastatic spread

**Figure 4:**
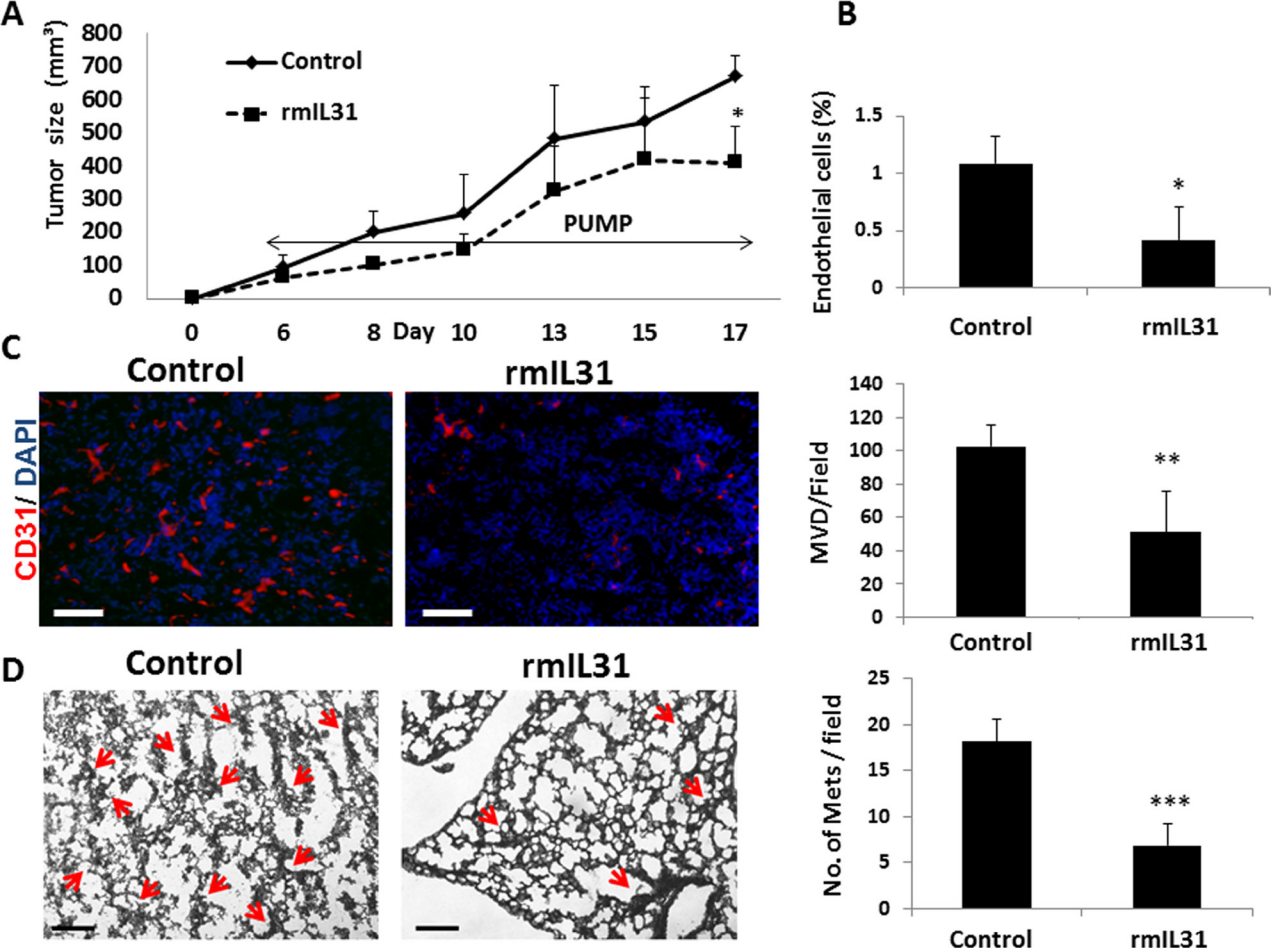
IL31 inhibits angiogenesis and lung metastasis in 4T1 metastatic breast carcinoma 4T1 cells (0.5 × 10^6^) were implanted into the mammary fat pad of 8 week old BALB/c mice (*n* = 5 mice/group). After 3 days, mice were implanted with micro-osmotic pumps containing rmIL31 (administered at a dose of 0.7 μg/day) or PBS (control). Tumor growth was assessed regularly (**A**). At end point, tumors were removed and divided into two equal parts. One part was sectioned (**B**) and the other part was prepared as a single cell suspension (**C**). Tumor sections were immunostained for CD31, an endothelial cell marker (red). Nuclei were stained with DAPI (blue). Scale bar = 200μm (B). Tumor single cell suspensions were assessed for the percentage of endothelial cells using flow cytometry (C). (**D**) Lungs were harvested at end point. Left panel: Lung sections were stained with H&E to visualize metastatic lesions (red arrows). Representative images are shown. Scale bar = 200 μm. Right panel: Metastatic lesions per field were quantified (*n* > 15 fields/group) *p < 0.05; **, 0.01 > *p* > 0.001; ***p < 0.001.

Angiogenesis has a potent effect on metastasis [24]. In light of our findings demonstrating that IL31 has an antiangiogenic effect, we next evaluated whether IL31 inhibits metastasis. To this end, we used the highly metastatic 4T1 murine breast carcinoma cell line which generates spontaneous pulmonary metastasis [25]. The 4T1 cell line highly expresses IL31RA, but not IL31 (Figure 1A). 4T1 cells were implanted to the mammary fat pad of BALB/c mice. After 3 days, mice were implanted with micro-osmotic pumps and infused with rmIL31 (0.7 μg/day for 2 weeks). Tumor growth, percentage of endothelial cells and MVD in tumors were significantly reduced in mice infused with IL31 compared to control mice (Figure 4A–4C), similar to our findings with MC38 tumors. In addition, a significant decrease in the number of micrometastases was observed in the lungs of mice infused with IL31 compared to control mice (Figure 4D). These results demonstrate that IL31 inhibits both angiogenesis and pulmonary metastasis.

### IL31 directly inhibits endothelial cell tube formation

**Figure 5:**
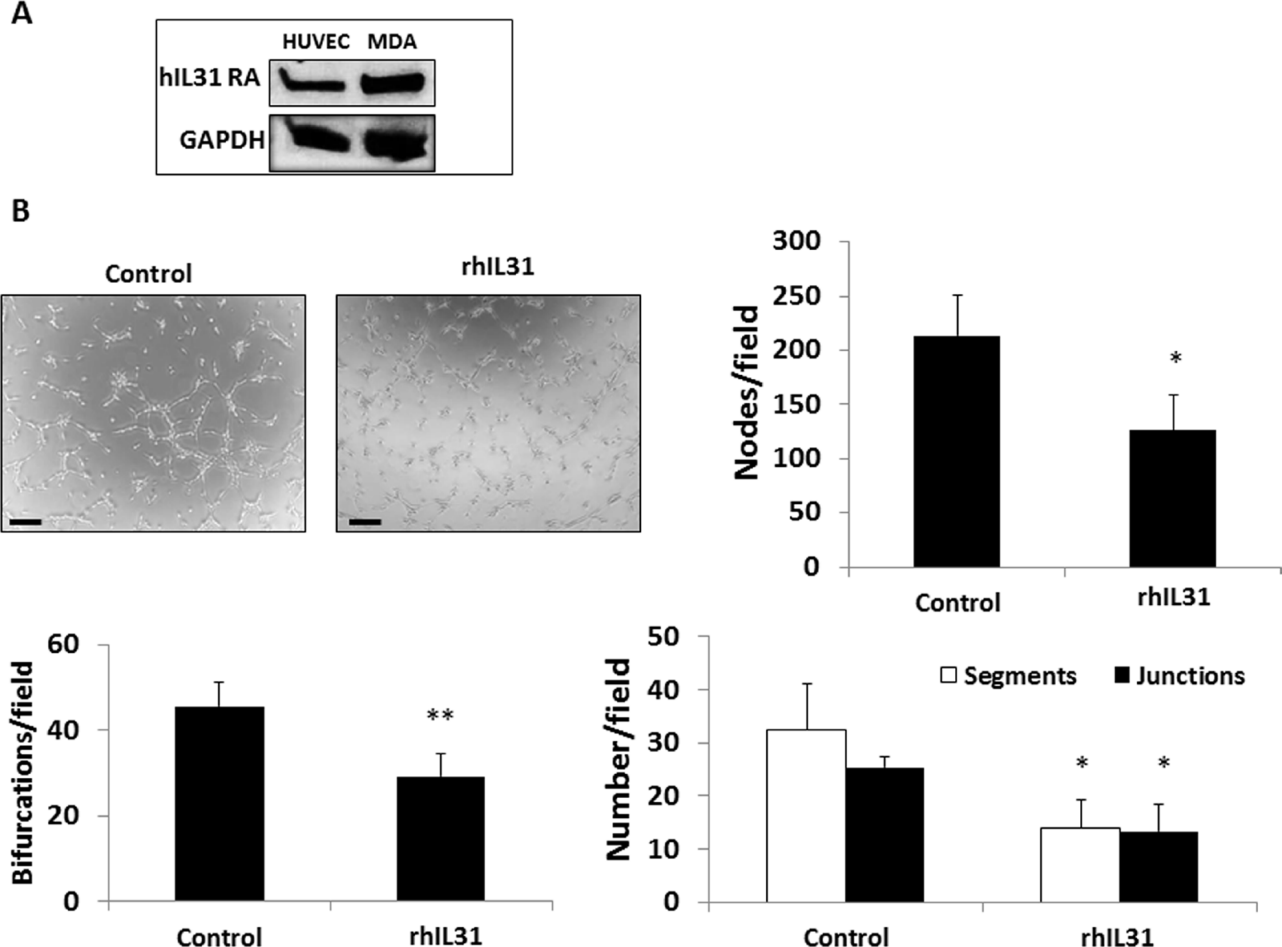
IL31 directly inhibits endothelial cell tube formation (**A**) The expression level of IL31RA was evaluated in lysates of HUVECs and MDA-MB-231(MDA) cells (positive control) by Western blot. (**B**) HUVECs were cultured in Matrigel-coated plates and treated with vehicle control or rhIL31 (100 ng/ml) for 24 hours. The formation of tubes was monitored over time using a time-lapse inverted wide-field microscope (Zeiss Axio observer microscope, Germany). Representative images of the 80 min time-point are shown. Scale bar = 250 μm. The number of bifurcations, nodes, segments, and junctions per field (*n* = 5 fields/group) were analyzed using ImageJ software, and plotted (**C**). *p < 0.05; **, 0.01 > *p* > 0.001.

We next asked whether IL31 directly affects endothelial cell activity. We first examined the expression of IL31RA on endothelial cells and found that human umbilical vein endothelial cells (HUVECs) indeed express IL31RA (Figure 5A). Next we performed a HUVEC tube forming assay in the presence of rhIL31 or PBS control. IL31 significantly delayed the formation of tubes, as demonstrated by a significant reduction in the number of bifurcations, nodes, segmentations and junctions in the presence of IL31 compared to control (Figure 5B). These results suggest that IL31 directly inhibits HUVEC tube forming activity.

### IL31-IgG fusion protein is active and stable in the circulation

Small proteins, such as cytokines and chemokines, have a short half-life in peripheral blood circulation, usually in the range of several minutes [26, 27] such that their use as therapeutic agents is not feasible in a clinical setting. To increase the stability of IL31, we designed a construct encoding IL31 fused to the CH2-CH3 domain of the IgG backbone. This fusion presumably increases the half-life of the protein as previously shown for other molecules [28, 29]. HEK-293T cells were transfected with the human IL31-IgG expression plasmid and stable transfectants were selected. Expression of the fusion protein was detected in cell lysates by Western blot analysis using appropriate antibodies (Supplementary Figure 3A–3B). For purification of IL31-IgG, the cells were serum-starved for 72 hours and conditioned medium was run on nickel-bead columns.

To evaluate the stability of the purified IL31-IgG protein in peripheral blood, C57/Bl6 mice were injected with protein mixture containing 200 μg of the recombinant human IL31-IgG protein together with 30 μg of a recombinant human IL31 protein tagged with His. Blood was drawn from the retro-orbital sinus at different time points and plasma was separated. The IL31 proteins were detected by Western blot analysis using anti-His HRP conjugated antibody. While the recombinant IL31 was no longer detected 15 min after injection, the IL31-IgG fusion protein remained in the circulation for at least 72 hours (Supplementary Figure 3C).

We next verified that the purified IL31-IgG protein is functionally active by testing its ability to activate the STAT3 pathway. To do this, U87 human glioblastoma cells expressing IL31RA (Figure 1B) were transfected with a STAT3 luciferase reporter plasmid. Cells were then serum starved for 16 hours followed by a 6 hour incubation with IL31-IgG or rhIL31 proteins. Both proteins significantly activated the STAT3 pathway, although a higher concentration of IL31-IgG was required to achieve a comparable level of activation to that found with rhIL31 (Supplementary Figure 3D). Overall, these results demonstrate that the IL31-IgG fusion protein is functionally active and is stable in the circulation for at least 72 hours.

### IL31-IgG inhibits tumor growth, angiogenesis and metastasis

**Figure 6:**
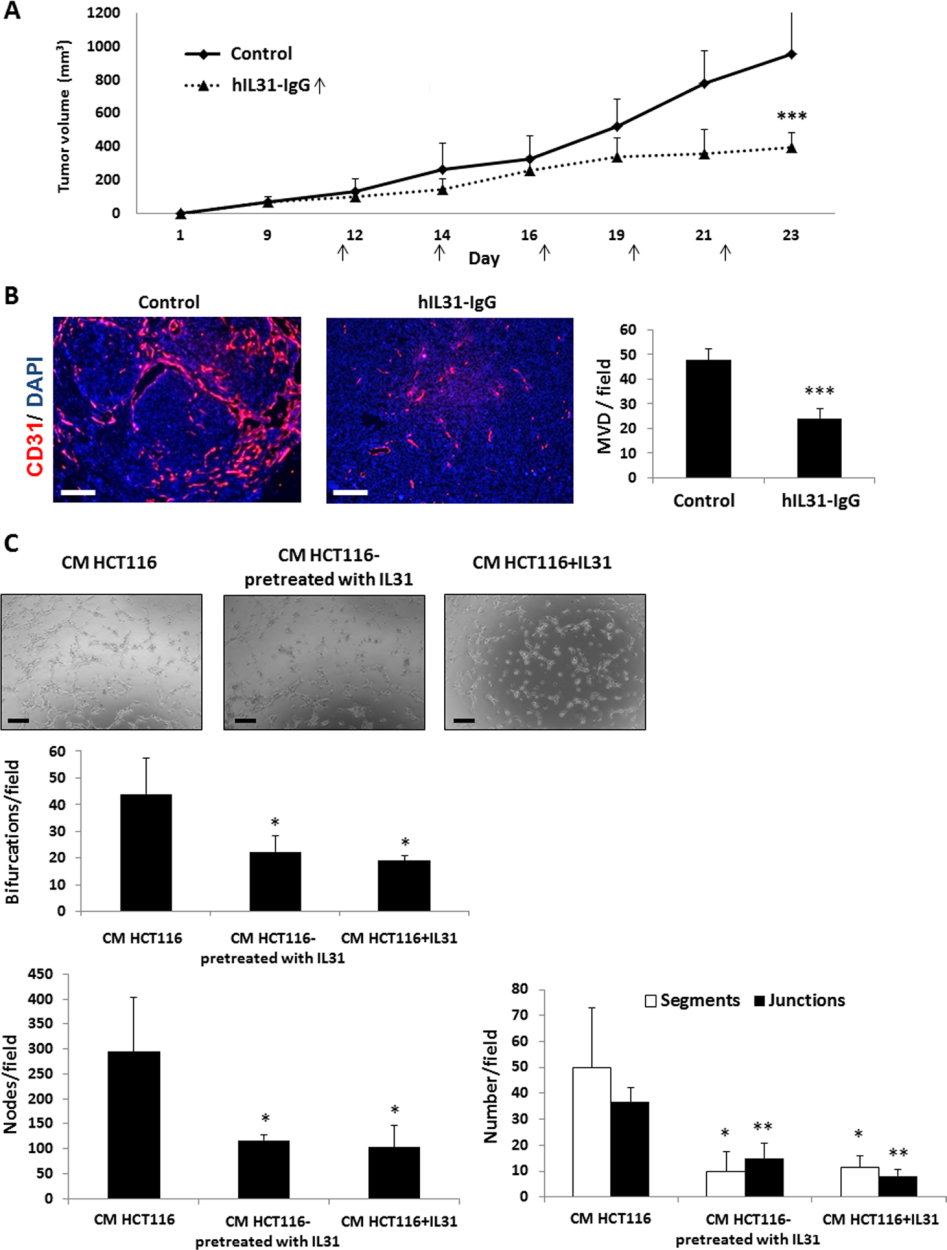
Human IL31-IgG inhibits tumor growth and angiogenesis (**A**–**B**) HCT116 human colon carcinoma cells (2 × 10^6^) were subcutaneously implanted into the flanks of NOD-SCID mice (*n* = 5 mice/group). When tumors reached a size of 100 mm^3^, mice were intraperitoneally injected twice weekly with 50 μg hIL31-IgG, or IgG control, and tumor growth was assessed over time (A). Tumors were removed at end point and sectioned. Microvessel density was evaluated by immunostaining with anti-CD31 antibodies (red). Nuclei were stained with DAPI (blue). The number of microvessels per field (*n* > 15 fields/group) was quantified (B). Scale bar = 200 μm (**C**) HUVECs were cultured with conditioned medium (CM) obtained from HCT116 cultures that were previously treated with vehicle control or 100 ng/ml rhIL31. As a positive control, rhIL31 (100 ng/ml) was added to control CM. The formation of tubes was monitored over time using a bright-field time-lapse microscope. Representative images of the 80 min time-point are shown. Scale bar = 250 μm. The number of bifurcations, nodes, segments, and junctions per field (*n* = 5 fields/group) were analyzed using ImageJ software, and plotted. *, 0.05 > *p* > 0.01; **, 0.01 > *p* > 0.001; ***p < 0.001.

We next investigated the effect of IL31-IgG in human models of cancer using NOD-SCID mice. These mice are deficient of innate and adaptive immune cell components and therefore any potential immunomodulation effects of the IL31-IgG protein can be ruled out in this system [30]. HCT116 human colon carcinoma cells were subcutaneously implanted into the flanks of NOD-SCID mice. When tumors reached 100 mm^3^, mice were intraperitoneally injected with IL31-IgG twice weekly, and tumor growth was assessed over time. A significant inhibition of tumor growth was found in IL31-IgG-treated mice compared to control IgG-treated mice (Figure 6A). In addition, MVD count was decreased in tumors of IL31-IgG-treated mice (Figure 6B). However, vessel structure was not markedly changed in contrast to results shown in Figure 3E.

It should be noted that the human IL31-IgG does not cross react with IL31RA found in the mouse endothelial cells, due to only ∼30% homology between human and mouse IL31 [14] and data not shown. Therefore, it is plausible that IL31 inhibits endothelial cells indirectly by inducing the secretion of antiangiogenic mediators from human tumor cells. To test this possibility, HCT116 colon carcinoma cells that had been previously cultured in the presence of rhIL31 were subsequently cultured in serum-free medium to generate conditioned medium (CM). Then, HUVECs were exposed to CM in order to evaluate endothelial cell activity. The CM from cells pre-treated with rhIL31 reduced tube formation in comparison to the corresponding control demonstrating an indirect effect of human IL31 on mouse endothelial cell activity (Figure 6C, middle panels). In addition, supplementing IL31 to CM from cells pre-treated with control IgG similarly reduced tube formation (Figure 6C, right panels) in accordance with results shown in Figure 5. Collectively, our findings demonstrate that IL31 inhibits endothelial cell activity both directly (Figure 5) and indirectly probably through antiangiogenic mediators secreted by tumor cells (Figure 6C).

**Figure 7:**
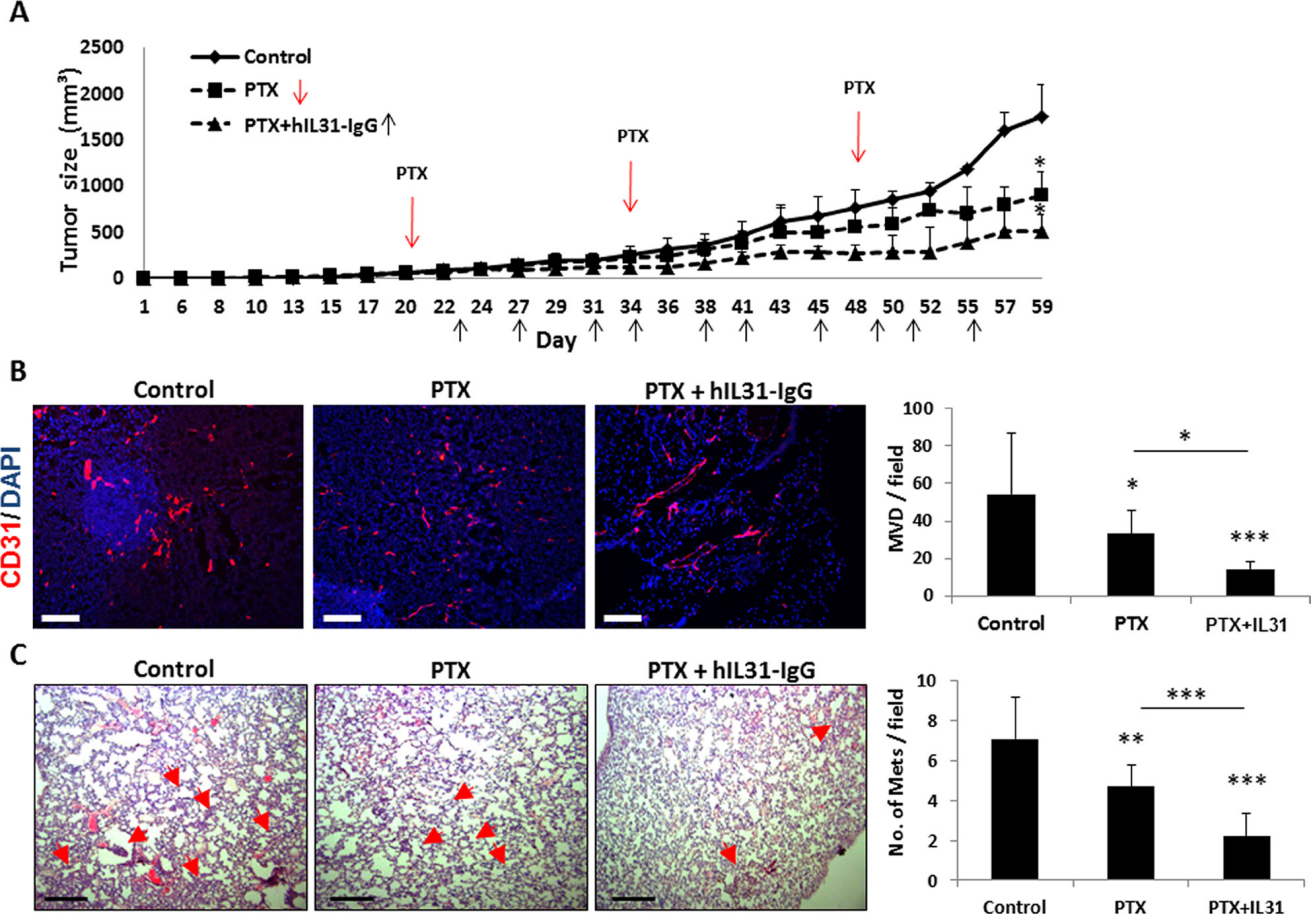
IL31 therapy enhances the anti-cancer activity of paclitaxel chemotherapy LM2-4 human breast carcinoma cells (2 × 10^6^) were implanted into the mammary fat pad of 6–8 week old NOD-SCID mice (*n* = 4–5 mice/group). When tumors reached a size of 50 mm^3^, treatment with paclitaxel (PTX) or PTX+IL31-IgG was initiated. (**A**) Tumor growth was assessed over time. (**B**) Tumors were removed at end point and sectioned. Tumor sections were immuno-stained with CD31 (red). Nuclei were stained with DAPI (blue). Scale bar = 200 μm. Microvessels per field (*n* > 15 fields/group) were quantified. (**C**) Lungs were removed and sectioned. Lung sections were stained with H&E to visualize metastatic lesions (red arrows). Representative images are shown. Scale bar = 200 μm. Metastatic lesions per field were counted (*n* > 10 fields/group) *,0.05 > *p* > 0.01; **, 0.01 > *p* > 0.001; ***p < 0.001.

Lastly, we evaluated the effect of IL31-IgG on metastasis in a clinically relevant breast carcinoma model. In this case, IL31 was used as an ‘add-on’ drug in combination with standard chemotherapy. It has been shown that angiogenesis is induced between successive chemotherapy treatments and therefore the addition of an antiangiogenic drug may increase the efficacy of chemotherapy [8, 31]. Clinically, antibody-based antiangiogenic drugs are usually administered in combination with chemotherapy as they have little effect as single agents [32]. To test this treatment protocol in our preclinical model, LM2-4 human breast carcinoma cells were orthotopically implanted into the mammary fat pad of NOD-SCID mice. When tumors reached 50 mm^3^, treatment with PTX chemotherapy administered at the maximum tolerated dose (25 mg/kg) was initiated with or without IL31-IgG injections administered twice weekly. The combined therapy exhibited a superior anti-tumor activity in comparison to PTX monotherapy (Figure 7A). At end point, tumors and lungs were removed. Tumors from mice treated with the combination therapy exhibited a significant reduction in MVD in comparison to tumors from untreated and PTX-treated mice (Figure 7B). In addition, the number of pulmonary micrometastases was significantly reduced in mice treated with PTX and IL31 when compared to PTX monotherapy or control (Figure 7C). It should be noted that no signs of toxicity was observed as assessed by changes in body weight in this model as well as the model presented in Figure 3D (Supplementary Figure 4). Collectively, these results suggest that IL31 has a superior therapeutic activity when administered as an ‘add on’ drug in combination with chemotherapy.

## DISCUSSION

The tumor microenvironment is composed of malignant and host cells that interact via an extended network of cytokines, chemokines, growth factors, and matrix remodeling enzymes [33, 34]. Such cells and factors are known to mainly promote but sometimes restrict tumor growth [3]. Recent studies have emphasized that following therapy, dramatic changes occur in the tumor microenvironment that may contribute to tumor re-growth and metastasis [1]. In this study we focused on the pro-inflammatory cytokine, IL31, which we previously found to be induced in response to chemotherapy. We found that IL31 and its receptor IL31RA are differentially expressed by various tumor types from both human and mouse origins. While IL31 has been shown to contribute to various inflammatory diseases such as dermatitis, inflammatory bowel disease, and airway hypersensitivity [35], in cancer, its role is still unknown. Importantly, while there is no clear correlation between autoimmune disease such as dermatitis and overall cancer incidence, based on Kaplan-Meier plotter for breast cancer patients, we found that there is a significant extended survival to breast cancer patients with high expression of IL31RA when compared to patients with low expression of IL31RA [36]. In addition, IL31 is highly expressed in hematological malignancies such as follicular lymphoma, germinal cancer derived B-cell malignancy, and T cell lymphoma, and was found to contribute to tumor growth via the STAT1/STAT3 signaling pathway [37]. On this note, unlike solid tumors in which pro-inflammation at the time of tumor progression usually restrict tumor growth [38], in hematological malignancies pro-inflammation promotes tumor growth as recently reported for multiple myeloma [39]. In this study, however, we focused on the non-immunological effects of IL31 on growth and metastasis of solid tumors.

We show that MC38 cells depleted of IL-31 exhibit an increase in invasive and migratory properties *in vitro*, effects that are reversed by supplementing the cells with exogenous IL-31. *In vivo*, the number of metastatic lesions in the lungs of 4T1 tumor-bearing mice is reduced when the mice are infused with IL31, in agreement with our *in vitro* findings. These anti-metastatic effects can be explained by the anti-angiogenic effects of IL31 and by the decreased invasion and migration properties of tumor cells in the presence of IL31. We also show that endothelial cells cultured in the presence of IL31 exhibit limited tube forming capability, including a reduction in the number of bifurcations, segments and nodes. Taken together, it is plausible that IL31 inhibits cell motility. Indeed, the effect of pro-inflammatory cytokines on cell motility and metastatic properties has already been reported. For example IL-1β induces the expression of metalloproteinase such as MMP9, and thus contributes to cell invasion [40]. TNFα and IFNγ have also been shown to promote motility of neural precursors [41]. It is plausible that IL31, like other pro-inflammatory cytokines, alters tumor cell activity by inhibiting cell motility rather than increasing it, explaining its inhibitory effect on angiogenesis and metastasis.

Tumor growth is highly dependent on angiogenesis [42]. Antiangiogenic therapy is a standard treatment for various malignancies, including colon, ovarian, non-small cell lung and glioblastoma tumors, among others [32]. Here we demonstrate that tumors from mice infused with IL31 exhibit reduced angiogenesis when compared to control untreated mice in both syngeneic murine tumors and human tumor xenografts implanted in NOD-SCID mice. Furthermore, HUVEC tube formation is inhibited in the presence of IL31 or CM of cells pre-treated with IL31, demonstrating both direct and indirect inhibition of endothelial cell activity, respectively. It is possible that IL31 induces the secretion of additional cytokines and factors as recently summarized in [35], which in turn inhibit the secretion of pro-angiogenic factors by tumor cells. In contrast, a recent study demonstrated that IL31 in conjunction with IL-4 induces the expression of VEGF and EGF in bronchial epithelial cells thereby supporting bronchial inflammation [43]; however, its direct angiogenic role and the relevant factors that are upregulated in response to IL31 activated signaling pathways have not been elucidated in carcinomas. Collectively, while the antiangiogenic activity of IL31 has been demonstrated *in vitro* and *in vivo*, it remains to evaluate whether IL31 affects also subsets of endothelial cells, e.g., tumor associated endothelium, therefore explaining its potent antiangiogenic activity *in vivo*.

To test the potential therapeutic effect of IL-31 in human-relevance cancers, we used IL-31-IgG fused protein with extended half-life and demonstrate its therapeutic potential *in vivo* when used in combination with chemotherapy. The rationale for using a combinatorial approach is based on previous reports indicating that chemotherapy and other cytotoxic-like agents stimulate neoangiogenesis ultimately leading to therapy resistance and tumor re-growth [7, 44]. Accordingly, the addition of an anti-angiogenic drug in between successive chemotherapy treatments blocks the rapid neoangiogenesis process, enhancing therapy outcome [8, 45]. In this study we show that injecting tumor-bearing, chemotherapy-treated mice with IL31-IgG reduces tumor growth, angiogenesis and pulmonary metastases to a greater extent than when chemotherapy is used alone. We also found that treated mice did not exhibit toxic side effects such as dermatitis often found with overexpression of IL31 [18] and there were no changes in body weight during the therapy. These findings highlight the potential use of IL31 as an anti-cancer drug. We should note that the use of NOD-SCID mice, which are deficient of immune cells, allowed us to rule out the cancer associated inflammation that may take place following IL31 therapy inflammation.

In conclusion, this study demonstrates the anti-tumorigenic effect of IL31 that is mediated by inhibition of angiogenesis and metastasis. However, additional studies are required to assess the pro-inflammatory and immunomodulatory effects of IL31 in the tumor microenvironment, and whether such activities affect tumorigenesis.

## MATERIALS AND METHODS

### Cell culture

MC38 murine colon carcinoma, EMT6 murine breast carcinoma, B16 murine melanoma, K7M2 murine osteosarcoma, LLC1 murine Lewis lung carcinoma, MDA-MB-231 and MCF-7 human breast carcinomas, SW480 and HCT116 human colon carcinomas, PANC1 human pancreatic ductal adenocarcinoma, and U87 human glioblastoma cell lines were cultured in Dulbecco’s modified eagle’s medium (DMEM, Sigma-Aldrich, Israel) supplemented with 10% fetal calf serum (FCS, Biological Industries, Israel), 1% L-glutamine, 1% sodium pyruvate, and 1% streptomycin-penicillin-neomycin solution (Biological Industries). MC38 was kindly provided by Prof. Ron Apte (Ben Gurion university, Israel), and all other indicated cell lines were obtained from the American Type Culture Collection (ATCC, Manassas, VA, USA) and were used within 6 months of cell resuscitation. 4T1 murine breast carcinoma, CT-26 murine colon carcinoma, P3 and MPC-11 murine myelomas, LM2-4 human breast carcinoma, and Jurkat human acute T-cell leukemia cell lines, all from ATCC, were cultured in RPMI-1640 medium (Sigma-Aldrich) supplemented as above. Human umbilical vein endothelial cells (HUVECs) were obtained from Lonza (Basel, Switzerland) and were cultured in M199 medium (Biological Industries) supplemented with 20% FCS, 2 mM glutamine, 1% streptomycin, penicillin and neomycin, MEM vitamins (1:100), and bFGF (5 ng/ml, Biological Industries). All cells were cultured at 37°C in a humidified atmosphere containing 5% CO_2_.

### Tumor models

MC38 (1 × 10^6^) or HCT116 (2 × 10^6^) cells were subcutaneously injected into the flanks of 6-week-old female C57Bl/6 or non-obese diabetic severe combined immunodeficient (NOD-SCID) mice (Harlan, Israel), respectively. 4T1 (5 × 10^5^) and LM2-4 (2 × 10^6^) cells were orthotopically injected into the mammary fat pad of 6–8 week-old female BALB/c or NOD-SCID female mice (Harlan, Israel), respectively. Tumor size was assessed regularly with Vernier calipers using the formula width^2^ × length × 0.5. All animal studies were performed in accordance with the Animal Care and Use Committee of the Technion-Israel Institute of Technology.

### Preparation of cell lysates and Western blot analysis

Cells were lysed in cell lysis buffer (25 mM HEPES pH 7.7, 0.3 M NaCl, 1.5 mM MgCl_2_, 0.2 mM EDTA, 1% Triton X-100), supplemented with 0.5 mM DTT, 20 mM β-glycerophosphate, 0.1 mM Na_2_VO_4_, 100 μg/ml PMSF, and protease inhibitor cocktail 1:100 (Sigma Aldrich). Samples were centrifuged at 10,000 g for 10 min at 4^°^C. Protein concentration was determined by Bradford. Sample buffer was then added to the cell lysates. One hundred micrograms of protein was separated by SDS/PAGE. Subsequently, the samples were electro-transferred to nitrocellulose membranes. The membranes were blocked in 5% skim milk in TBST for 30 min followed by overnight incubation at 4°C with the following antibodies: anti-mouse IL31 (5 μg/ml, Abcam, Cambridge, UK), anti-mouse IL31RA (1:100, BIOSES) or anti-human IL31RA (5 μg/ml, Sigma Aldrich). Actin mouse monoclonal antibody (1:5000, MP Biomedicals) or GAPDH (1: 500, Santa Cruz Biotechnology, Inc.) were used to verify equal loading. Proteins were detected by enhanced chemiluminescence (Biological Industries).

### mRNA extraction and quantitative RT-PCR

mRNA was purified from the various human cell lines using High Pure RNA Isolation Kit (Roche Diagnostics, Manheim, Germany) in accordance with the manufacturer’s protocol. mRNA was quantified by measuring Ab260 nm with a Nano Drop spectrophotometer (ND-1000, Nano Drop Technologies, Rockland, DE, USA). cDNA was then synthesized from the mRNA samples (100–1000 ng of mRNA in a 20 μl total reaction mix) using a high capacity cDNA reverse transcription kit (iScriptTMcDNA Synthesis Kit, Bio-Rad Laboratories Inc.). Real-time PCR for human IL31 was performed using Rotor-Gene 6000TM (Corbett) equipment with iTaqTM universal probes supermix (iTaqTMUniversal Probes Supermix, Bio-Rad Laboratories Inc.) using hIL31 probe (#Hs01098710_m1, Thermo Fisher Scientific Inc.). Values were normalized to HPRT (hypoxanthine phosphoribosyltransferase) (#Hs02800695_m1, Thermo Fisher Scientific Inc.) expression levels. Calculations of Taqman-analyzed transcripts were presented by semi-quantitative PCR.

### Downregulation of IL31 by siRNA

Four different siRNA sequences specific to murine IL31 or a scrambled control sequence were cloned into the piLenti-siRNA-GFP plasmid (Applied Biological Materials, Richmond, BC, Canada). Subconfluent MC38 cells were transfected with the siRNA plasmids using FuGENE^®^ 6 transfection reagent (Roche, Penzberg, Germany) according to the manufacturer’s instructions. Forty-eight hours post-transfection, cells were incubated in growth medium containing puromycin (1 μl/ml) for the selection of stable transfectants. After 2 weeks of selection, the protein level of IL31 was evaluated by Western blot.

### Invasion and migration assays

The invasion and migration properties of tumor cells were evaluated in Matrigel- or fibronectin-coated Boyden chambers as previously described [4]. Briefly, filters (6.5 mm in diameter, 8 μm pore size) were coated with either Matrigel (diluted 1:4 in DMEM, BD Bioscience, USA), or 0.01 μg/μl fibronectin (Biological Industries, Israel) for invasion or migration assays, respectively. Freshly coated filters were left to dry for 2 hours at 37°C. Serum-deprived siIL31 or control MC38 cells were seeded in the upper compartment of the chamber and the lower compartment was filled with serum-free medium. After incubation for 24 hours at 37°C, cells that passed through to the bottom filter were fixed with 4% paraformaldehyde (PFA). Cells were stained with 0.5% Crystal violet (Sigma-Aldrich) and visualized using Leica DMI 6000B microscope (Leica, Wetzlar Germany). All experiments were performed in triplicate.

### Cell viability alamar blue^TM^ assay

Cell viability was evaluated quantitatively with the metabolic indicator dye AlamarBlue^TM^ (Serotec Ltd., Oxford, UK), which determines the metabolic activity of cells, as previously described [46]. Briefly, MC38 or CT-26 cells were harvested from sub-confluent cultures and re-plated (500– 1,000 cells/well in a 96-well plate) in their designated medium supplemented with 10% AlamarBlue (AB) solution. Recombinant murine IL31 (rmIL31, PeproTech, Israel) was added in escalating concentrations (0.5–100 ng/ml). Absorbance was acquired daily using ELISA reader (TECAN infinite M200Pro, Switzerland). The percent of AB reduction was calculated using an appropriate equation. Results were corrected to background values of negative controls. All experiments were performed in triplicate.

### Cell cycle assay

MC38 or CT-26 cells were cultured with 100 ng/ml rmIL31 or vehicle control for 48 hours. Cells were detached and re-suspended in 100 μl PBS. Cells were fixed by slowly adding the suspension to a tube containing 1 ml 70% ethanol with gentle vortexing. Cells were incubated on ice for 20 min, centrifuged and re-suspended in 1ml of Propidium Iodide (PI) master mix containing 40 μg/ml PI (BioLegend, Inc., USA), 10 μg/ml RNase (Sigma-Aldrich), and 950 μl PBS. After a 30 min incubation at room temperature, samples were analyzed with a Cyan-ADP flow cytometer and Summit Version 4.3 software (Beckman Coulter, Switzerland) using linear scale histogram.

### Apoptosis assay

MC38 or CT-26 cells were cultured with 100 ng/ml rmIL31 or vehicle control for 48 hours. The cells were detached and re-suspended in 60 μl Annexin V Binding Buffer (MBL, Japan). Subsequently, 2.5 μl of Annexin V-FITC antibody (Bio Legend, Inc., USA) and 2.5 μl of 7-Aminoactinomycin D (7-AAD) (Sigma-Aldrich, USA) were added and samples were incubated for 15 min at RT in the dark. Annexin V Binding Buffer (300 μl) was then added to each sample. Samples were analyzed with a Cyan-ADP flow cytometer and Summit Version 4.3 software (Beckman Coulter, Switzerland).

### BrdU assay

MC38 or CT-26 cells were cultured with rmIL31 (100 ng/ml) or vehicle control for 48 hours. Subsequently they were incubated for 2 hours with 11 μM bromodeoxyuridine (BrdU) labeling reagent (MBL) using CycLex Cellular BrdU ELISA kit (CycLex Co. Japan) followed by the manufacturer’s instruction. BrdU was detected by ELISA.

### Osmotic pumps

Alzet micro-osmotic pumps were used to achieve continuous administration of rmIL31 to mice for a period of 2 weeks, as previously described [47]. Briefly, C57Bl/6 tumor-bearing mice were subcutaneously implanted with micro-osmotic pumps (#1002, Alzet, Cupertino, CA) loaded with rmIL31 (at a dose of 0.7 μg/day) or PBS as a vehicle control. The procedure was performed under sterile conditions. The mice were sacrificed 14 days after pump implantation and further assessed as described in the text.

### Flow cytometry analysis of endothelial cells

Tumor specimens were prepared as single cell suspensions as previously described [48]. Cells from tumor specimens were immunostained with CD45-APC-Cy7, CD31-APC, and VEGFR2-PE (BioLegend, San Diego, CA). Endothelial cells were defined as CD45–/CD31+/VEGFR2+. At least 100,000 events were collected for each sample. The experiments were performed using Cyan-ADP flow cytometer and analyzed by Summit Version 4.3 software (Beckman Coulter, Switzerland).

### Tissue processing and immunostaining

Tumors and lungs were embedded in OCT and subsequently sectioned. Lungs were stained with H&E in order to detect metastatic lesions. Tumor sections (10 μm) were analyzed for microvessel density (MVD) by immunostaining using an anti-CD31 antibody (1:200, BD Biosciences, San Jose, CA, USA) followed by a Cy3-conjugated secondary antibody (1:500, (Jackson). Vessel structures per field were counted and plotted. At least 5 fields per tumor, normally *n* > 15 fields per group, were counted. Cancer survey tissue microarrays (TMA, OriGene Technologies, Inc., Rockville, MD USA) or tumor sections from human breast (*n* = 15) and colon (*n* = 15) carcinoma samples obtained from the Department of Pathology at the Rambam Medical Center (Haifa, Israel), were immunostained with human IL31RA antibody (1:100, Abcam) followed by histidine peroxidase anti-mouse and anti-rabbit secondary antibodies (Nichirei, Japan). Staining was developed using AEC simple stain solution (Nichirei). Hematoxylin (Sigma) was used as a counterstain. The samples were analyzed by a pathologist for positive staining. All human studies were approved by the ethic committee at the Rambam Medical Center after patients signed an informed consent.

### Tube forming assay

HUVECs (4 × 10^5^) were seeded in 48 well plates pre-coated with 150 μl of Matrigel. The cells were incubated for 24 hours with serum-free DMEM supplemented with 100 ng/ml rhIL31 or PBS control. In a different experiment, HCT116 cells were cultured in the presence of 100 ng/ml rhIL31 or PBS control. After 24 hours, the cells were rinsed with PBS and fresh serum-free medium was added. Conditioned medium (CM) was collected 24 hours later for use in the tube forming assay. HUVECs were incubated for 24 hours with serum-free DMEM supplemented with CM. The formation of microtubes by the HUVECs was monitored using time-lapse microscopy (Zeiss Axio observer system). The number of bifurcations, nodes, segments and junctions were counted per field (*n* > 5 fields/group), using computer image analysis (ImageJ, NIH). The experiments were performed in triplicate.

### Human IL31-IgG construct generation

The DNA sequence of mature human IL31 protein with its signal peptide for secretion was synthesized based on gBlocks Gene Fragment technology from IDT, and inserted into the NSPI expression vector (kindly provided by Prof. Gera Neufeld, Technion, Israel). Mouse IgG1 heavy chain (hinge-CH2-CH3) was cloned downstream to human IL31 and upstream to myc-His6. Briefly, total RNA was isolated from mouse spleen using RNeasy Mini Kit (Qiagen). Single-stranded cDNA was synthesized using M-MLV reverse transcriptase (Promega, WI) according to manufacturer’s instructions. Mouse IgG1 heavy chain (hinge-CH2-CH3) was amplified using PCR. The primers used were: sense (5- TACCGCTCGAGGTGCCCAGGGATTGTGGTTG-3) and antisense (5′-CGTTCGAATTTACCAGGAGAG TGGG -3). The NSPI-IL31–mIgG–myc-His construct was checked by restriction mapping and sequencing.

### Affinity chromatography

HEK-293T human embryonic kidney cells (ATCC) were transfected with the NSPI-IL31–mIgG–myc-His plasmid using the calcium phosphate method [49]. After 48 hours, the cells were cultured in growth medium supplemented with puromycin (1 μl/ml) to select for stable transfectants (HEK-293T-IL31). Expression of the fusion protein was verified by Western blot, as described below. For purification of the IL31-IgG fusion protein, HEK-293T-IL31 cells were serum starved for 72 hours. Conditioned medium was then collected and run on a HisTrap affinity column (GE Healthcare, Sweden) using the AKTA start protein purification system (GE Healthcare, Sweden). Imidazole (0.25 M) was used to elute the IL31-IgG protein. The purified protein sample was run on a buffer exchange column (Thermo Fisher Scientific Inc.) to exchange imidazole buffer for PBS. Glycerol was added to the protein sample to a final concentration of 10%, and aliquots were stored at –20°C until further use. IL31-IgG fusion protein product was tested to be endotoxin-free using endotoxin detection kit (Endozyme, Hyglos, Germany).

### Pharmacokinetic analysis of the IL31-IgG fusion protein

C57Bl/6 mice were intraperitoneally injected with a mixture of 30 μg of hrIL31 and 200μg of hIL31-IgG. Blood was drawn from the retro-orbital sinus at baseline and after 0.25, 1, 6, 24, 72 and 144 hours. The blood samples were centrifuged (3000 × g, 15 min) to separate plasma. Plasma samples (2 μl/lane) were resolved by SDS/PAGE gel, electrotransferred to a nitrocellulose membrane, and analyzed by Western blot for the presence of IL31 and IL31-IgG using anti-mouse IgG (1:2000, Jacksons), anti-myc (1:500, Santa Cruz), and anti-His mAb-HRP-DirecT (1:3000, MBL). Plasma immunoglobulin, which served as a loading control, was detected using anti-mouse IgG light chain (1:10,000, Jackson).

### Luciferase reporter assay

U87 cells, highly expressing IL31RA, were transfected with a STAT3-luciferase reporter plasmid (kindly provided by Prof. Jian Jian Li, as previously described [50]) using lipofectamine reagent (Thermo Fisher Scientific, MA). Subsequently, cells were serum-starved for 16 hours, and then cells were either left untreated or treated with hrIL31 (50 ng/ml), IL31-IgG (5 μg/ml) or IgG (5 μg/ml) for an additional 6 hours. Cells were harvested, centrifuged and washed with PBS. The pellet was re-suspended in lysis buffer (Passive lysis buffer, Promega), and lysed by instant freeze/thaw cycles. Lysates were centrifuged and supernatants were tested for luciferase activity using the dual luciferase assay kit (Dual-Luciferase Reporter Assay System, Promega) according to the manufacturer’s instructions. Bioluminescence was quantified using the TD 20/20 luminometer (Turner Designs, Sunnyvale, CA, USA).

### Statistical analysis

Data are presented as mean ± standard deviation (SD). Statistically significant differences were determined by two-tailed Student’s *t* test or one-way ANOVA, followed by Tukey ad hoc statistical post-test using Prism 5 software (GraphPad, La Jolla, CA). Differences between all groups were compared with each other or to control (in the case of Student’s *t*-test), and were considered significant at values of *p < 0.05, **p < 0.01, and ***p < 0.001.

## SUPPLEMENTARY MATERIALS FIGURES



## References

[1] Shaked Y (2016). Balancing efficacy of and host immune responses to cancer therapy: the yin and yang effects. Nat Rev Clin Oncol.

[2] Katz OB, Shaked Y (2015). Host effects contributing to cancer therapy resistance. Drug Resist Updat.

[3] Hanahan D, Coussens LM (2012). Accessories to the crime: functions of cells recruited to the tumor microenvironment. Cancer Cell.

[4] Gingis-Velitski S, Loven D, Benayoun L, Munster M, Bril R, Voloshin T, Alishekevitz D, Bertolini F, Shaked Y (2011). Host response to short-term, single-agent chemotherapy induces matrix metalloproteinase-9 expression and accelerates metastasis in mice. Cancer Res.

[5] Daenen LG, Roodhart JM, van Amersfoort M, Dehnad M, Roessingh W, Ulfman LH, Derksen PW, Voest EE (2011). Chemotherapy enhances metastasis formation via VEGFR-1-expressing endothelial cells. Cancer Res.

[6] Welford AF, Biziato D, Coffelt SB, Nucera S, Fisher M, Pucci F, Di Serio C, Naldini L, De Palma M, Tozer GM, Lewis CE (2011). TIE2-expressing macrophages limit the therapeutic efficacy of the vascular-disrupting agent combretastatin A4 phosphate in mice. JClinInvest.

[7] Shaked Y, Henke E, Roodhart JM, Mancuso P, Langenberg MH, Colleoni M, Daenen LG, Man S, Xu P, Emmenegger U, Tang T, Zhu Z, Witte L (2008). Rapid chemotherapy-induced acute endothelial progenitor cell mobilization: implications for antiangiogenic drugs as chemosensitizing agents. Cancer Cell.

[8] Shaked Y, Kerbel RS (2007). Antiangiogenic strategies on defense: on the possibility of blocking rebounds by the tumor vasculature after chemotherapy. Cancer Res.

[9] Shaked Y, Tang T, Woloszynek J, Daenen LG, Man S, Xu P, Cai SR, Arbeit JM, Voest EE, Chaplin DJ, Smythe J, Harris A, Nathan P (2009). Contribution of granulocyte colony-stimulating factor to the acute mobilization of endothelial precursor cells by vascular disrupting agents. Cancer Res.

[10] Taylor M, Billiot F, Marty V, Rouffiac V, Cohen P, Tournay E, Opolon P, Louache F, Vassal G, Laplace-Builhe C, Vielh P, Soria JC, Farace F (2012). Reversing resistance to vascular-disrupting agents by blocking late mobilization of circulating endothelial progenitor cells. Cancer Discov.

[11] Bruchard M, Mignot G, Derangere V, Chalmin F, Chevriaux A, Vegran F, Boireau W, Simon B, Ryffel B, Connat JL, Kanellopoulos J, Martin F, Rebe C (2013). Chemotherapy-triggered cathepsin B release in myeloid-derived suppressor cells activates the Nlrp3 inflammasome and promotes tumor growth. Nature Medicine.

[12] Voloshin T, Alishekevitz D, Kaneti L, Miller V, Isakov E, Kaplanov I, Voronov E, Fremder E, Benhar M, Machluf M, Apte RN, Shaked Y (2015). Blocking IL-1beta pathway following paclitaxel chemotherapy slightly inhibits primary tumor growth but promotes spontaneous metastasis. Mol Cancer Ther.

[13] Alishekevitz D, Svetlana Gingis-Velitski, Orit Kaidr-Person, Lilach Gutter-Kapon, Sandra D. Scherer, Ziv Raviv, Emmanuelle Merquiol (2016). Macrophage-induced lymphangiogenesis and metastasis following paclitaxel chemotherapy is regulated by VEGFR3 Cell Reports.

[14] Dillon SR, Sprecher C, Hammond A, Bilsborough J, Rosenfeld-Franklin M, Presnell SR, Haugen HS, Maurer M, Harder B, Johnston J, Bort S, Mudri S, Kuijper JL (2004). Interleukin 31, a cytokine produced by activated T cells, induces dermatitis in mice. Nat Immunol.

[15] Nobbe S, Dziunycz P, Muhleisen B, Bilsborough J, Dillon SR, French LE, Hofbauer GF (2012). IL-31 expression by inflammatory cells is preferentially elevated in atopic dermatitis. Acta Derm Venereol.

[16] Castellani ML, Salini V, Frydas S, Donelan J, Madhappan B, Petrarca C, Vecchiet J, Falasca K, Neri G, Tete S (2006). Interleukin-31: a new cytokine involved in inflammation of the skin. Int J Immunopathol Pharmacol.

[17] Chattopadhyay S, Tracy E, Liang P, Robledo O, Rose-John S, Baumann H (2007). Interleukin-31 and oncostatin-M mediate distinct signaling reactions and response patterns in lung epithelial cells. J Biol Chem.

[18] Bilsborough J, Leung DY, Maurer M, Howell M, Boguniewicz M, Yao L, Storey H, LeCiel C, Harder B, Gross JA (2006). IL-31 is associated with cutaneous lymphocyte antigen-positive skin homing T cells in patients with atopic dermatitis. J Allergy Clin Immunol.

[19] Singer EM, Shin DB, Nattkemper LA, Benoit BM, Klein RS, Didigu CA, Loren AW, Dentchev T, Wysocka M, Yosipovitch G, Rook AH (2013). IL-31 is produced by the malignant T-cell population in cutaneous T-Cell lymphoma and correlates with CTCL pruritus. J Invest Dermatol.

[20] Dambacher J, Beigel F, Seiderer J, Haller D, Goke B, Auernhammer CJ, Brand S (2007). Interleukin 31 mediates MAP kinase and STAT1/3 activation in intestinal epithelial cells and its expression is upregulated in inflammatory bowel disease. Gut.

[21] Uhlen M, Alm T, Lundberg E, Lindskog Bergstrom C, Ponten F, Tegel H, Nillson P, Hober S, von Feilitzen K, S A. (2005). The human protein atlas.

[22] Cornelissen C, Luscher-Firzlaff J, Baron JM, Luscher B (2012). Signaling by IL-31 and functional consequences. Eur J Cell Biol.

[23] Zhang W, Liu HT (2002). MAPK signal pathways in the regulation of cell proliferation in mammalian cells. Cell Res.

[24] Mahadevan V, Hart IR (1990). Metastasis and angiogenesis. Acta Oncol.

[25] Schmaus A, Sleeman JP (2015). Hyaluronidase-1 expression promotes lung metastasis in syngeneic mouse tumor models without affecting accumulation of small hyaluronan oligosaccharides in tumor interstitial fluid. Glycobiology.

[26] Le T, Leung L, Carroll WL, Schibler KR (1997). Regulation of interleukin-10 gene expression: possible mechanisms accounting for its upregulation and for maturational differences in its expression by blood mononuclear cells. Blood.

[27] Noronha IL, Niemir Z, Stein H, Waldherr R (1995). Cytokines and growth factors in renal disease. Nephrol Dial Transplant.

[28] Park HI, Yoon HW, Jung ST (2016). The Highly Evolvable Antibody Fc Domain. Trends Biotechnol.

[29] Sondermann P, Szymkowski DE (2016). Harnessing Fc receptor biology in the design of therapeutic antibodies. Curr Opin Immunol.

[30] Greiner DL, Shultz LD, Yates J, Appel MC, Perdrizet G, Hesselton RM, Schweitzer I, Beamer WG, Shultz KL, Pelsue SC (1995). Improved engraftment of human spleen cells in NOD/LtSz-scid/scid mice as compared with C.B-17-scid/scid mice. Am J Pathol.

[31] Gokmen-Polar Y, Miller KD (2008). Redefining the target again: chemotherapeutics as vascular disrupting agents?. Cancer Cell.

[32] Jayson GC, Hicklin DJ, Ellis LM (2012). Antiangiogenic therapy—evolving view based on clinical trial results. Nat Rev Clin Oncol.

[33] Hanahan D, Weinberg RA (2011). Hallmarks of cancer: the next generation. Cell.

[34] Grivennikov SI, Greten FR, Karin M (2010). Immunity, inflammation, and cancer. Cell.

[35] Zhang Q, Putheti P, Zhou Q, Liu Q, Gao W (2008). Structures and biological functions of IL-31 and IL-31 receptors. Cytokine Growth Factor Rev.

[36] Balazs G (2009). Kaplan-Meier Plotter. http://kmplot.com.

[37] Ferretti E, Tripodo C, Pagnan G, Guarnotta C, Marimpietri D, Corrias MV, Ribatti D, Zupo S, Fraternali-Orcioni G, Ravetti JL, Pistoia V, Corcione A (2015). The interleukin (IL)-31/IL-31R axis contributes to tumor growth in human follicular lymphoma. Leukemia.

[38] Coussens LM, Werb Z (2002). Inflammation and cancer. Nature.

[39] Beyar-Katz O, Magidey K, Ben-Tsedek N, Alishekevitz D, Timaner M, Miller V, Lindzen M, Yarden Y, Avivi I, Shaked Y (2016). Bortezomib-induced proinflammatory macrophages as a potential factor limiting anti-tumour efficacy. J Pathol.

[40] Voronov E, Shouval DS, Krelin Y, Cagnano E, Benharroch D, Iwakura Y, Dinarello CA, Apte RN (2003). IL-1 is required for tumor invasiveness and angiogenesis. ProcNatlAcadSciUSA.

[41] Ben-Hur T, Ben-Menachem O, Furer V, Einstein O, Mizrachi-Kol R, Grigoriadis N (2003). Effects of proinflammatory cytokines on the growth, fate, and motility of multipotential neural precursor cells. Mol Cell Neurosci.

[42] Folkman J (1972). Anti-angiogenesis: new concept for therapy of solid tumors. Ann Surg.

[43] Ip WK, Wong CK, Li ML, Li PW, Cheung PF, Lam CW (2007). Interleukin-31 induces cytokine and chemokine production from human bronchial epithelial cells through activation of mitogen-activated protein kinase signalling pathways: implications for the allergic response. Immunology.

[44] Shaked Y, Ciarrocchi A, Franco M, Lee CR, Man S, Cheung AM, Hicklin DJ, Chaplin D, Foster FS, Benezra R, Kerbel RS (2006). Therapy-induced acute recruitment of circulating endothelial progenitor cells to tumors. Science.

[45] Kerbel RS (2008). Tumor angiogenesis. New England Journal of Medicine.

[46] Voloshin T, Gingis-Velitski S, Bril R, Benayoun L, Munster M, Milsom C, Man S, Kerbel RS, Shaked Y (2011). G-CSF supplementation with chemotherapy can promote revascularization and subsequent tumor regrowth: prevention by a CXCR4 antagonist. Blood.

[47] Koren L, Alishekevitz D, Elhanani O, Nevelsky A, Hai T, Kehat I, Shaked Y, Aronheim A (2015). ATF3-dependent cross-talk between cardiomyocytes and macrophages promotes cardiac maladaptive remodeling. Int J Cardiol.

[48] Timaner M, Beyar-Katz O, Shaked Y (2016). Analysis of the Stromal Cellular Components of the Solid Tumor Microenvironment Using Flow Cytometry. Curr Protoc Cell Biol.

[49] Batard P, Jordan M, Wurm F (2001). Transfer of high copy number plasmid into mammalian cells by calcium phosphate transfection. Gene.

[50] Yu CY, Wang L, Khaletskiy A, Farrar WL, Larner A, Colburn NH, Li JJ (2002). STAT3 activation is required for interleukin-6 induced transformation in tumor-promotion sensitive mouse skin epithelial cells. Oncogene.

