# Supplementary material for: The antiangiogenic role of the pro-inflammatory cytokine interleukin-31
Source: Oncotarget. 2017 Jan 27;8(10):16430–44. doi: 10.18632/oncotarget.14857 (PMC5369974; doi:10.18632/oncotarget.14857)
Supplement: Supplementary file 1 [file oncotarget-08-16430-s001.pdf]

The antiangiogenic role of the pro-inflammatory cytokine interleukin-31

Supplementary Materials

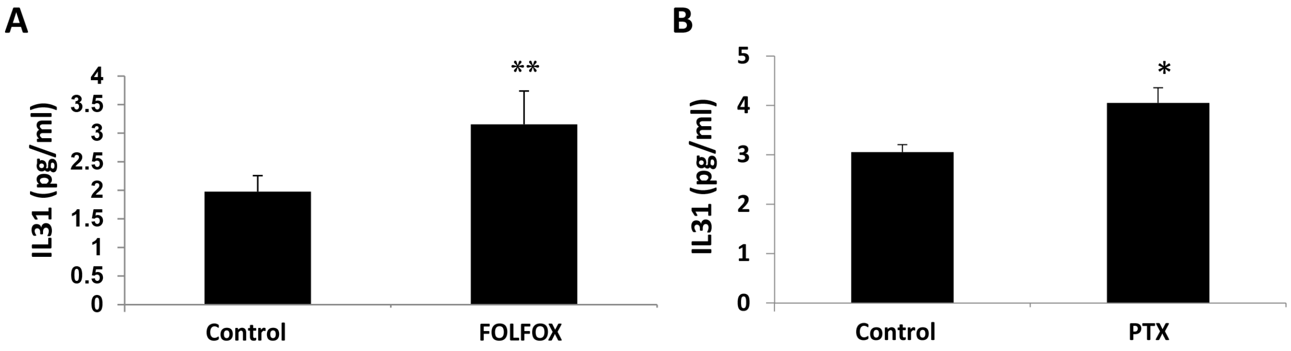

Supplementary Figure 1: Increased expression of IL31 in the plasma and bone marrow cells of mice treated with chemotherapy. Non tumor bearing BALB/c mice ($n = 4$) were treated with either maximum tolerated dose of folinic acid/5-fluorouracil/oxaliplatin (FOLFOX) or paclitaxel (PTX) chemotherapy. After 24 hours (**A**) bone marrow cells were flushed from the femurs or (**B**) blood was obtained by cardiac puncture. Cell lysates and plasma, respectively, were evaluated for IL31 expression using ELISA. *, $0.05 > p > 0.01$; **$p < 0.01$.

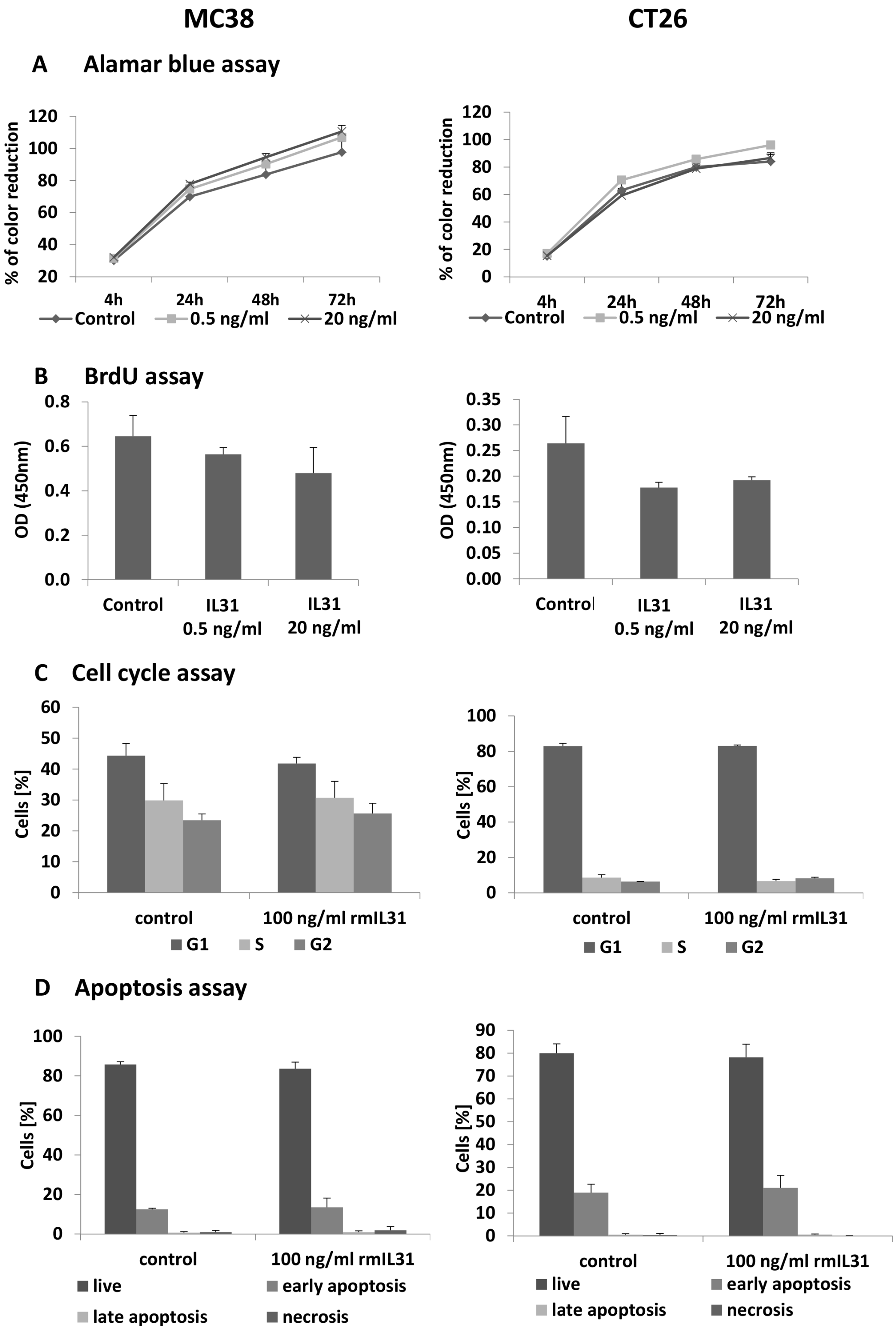

Supplementary Figure 2: IL31 does not affect cell viability, proliferation, cell cycle, and apoptosis. MC-38 and/or CT-26 murine colon carcinoma cells were evaluated for cell viability by Alamar Blue assay. (**A**) cell proliferation by BrdU (**B**) as well as cell cycle (**C**) and apoptosis (**D**) by flow cytometry. All assays are described in Materials and Methods. **, $0.01 > p > 0.001$; ***$p < 0.001$.

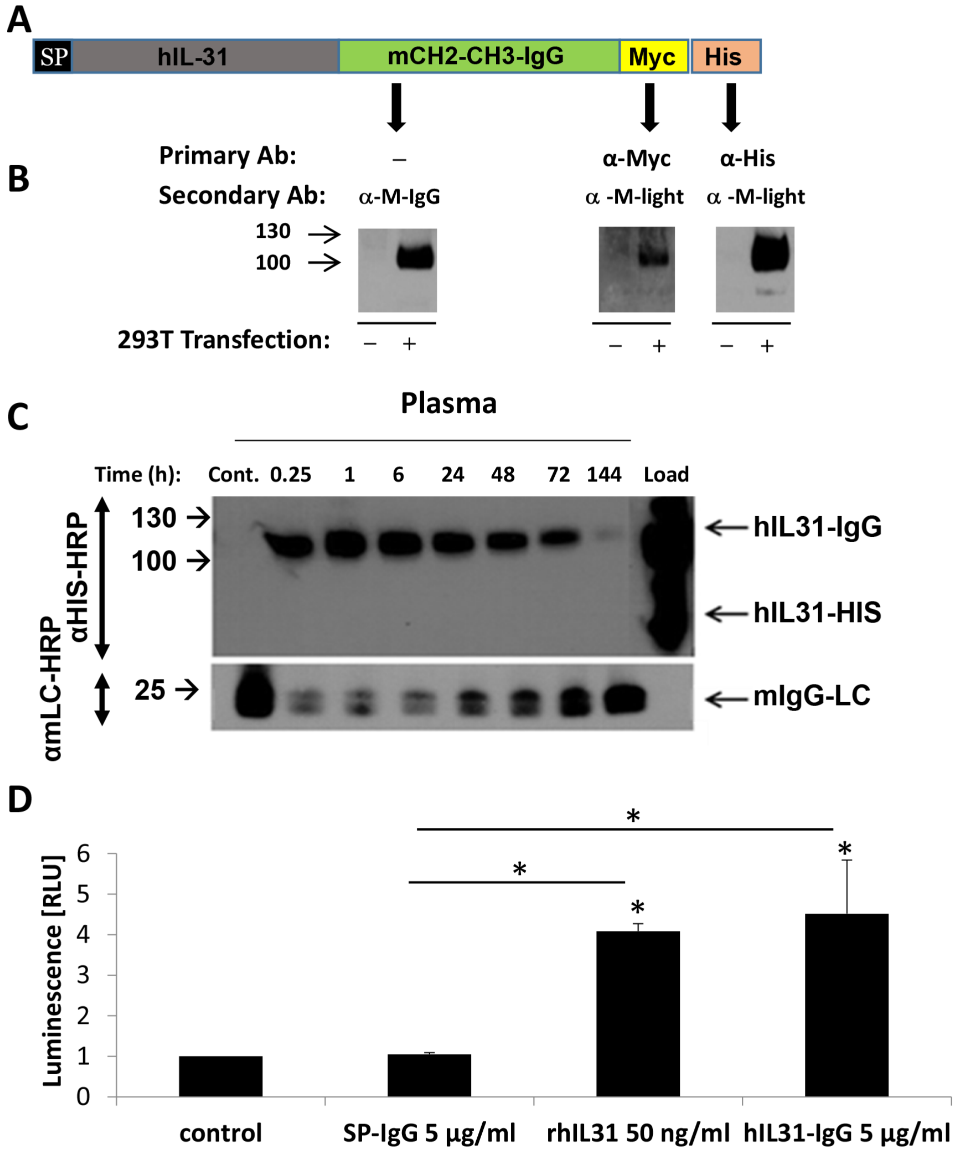

Supplementary Figure 3: Generation of stabilized form of IL31. (**A**) Illustration of the IL31-IgG construct: The DNA sequence of hIL31 protein with its signal peptide (SP) was synthesized and inserted into the NSPI expression vector. Mouse IgG1 heavy chain (hinge-CH2-CH3) was cloned downstream to hIL31 and upstream to myc-His6 tag. (**B**) HEK-293T cells were stably transfected with the IL31-IgG construct. Expression was verified by Western blot analysis using the indicated antibodies to identify the various components of the IL31-IgG fusion protein. (**C**) Eight-week old C57Bl/6 mice were intraperitoneally injected with a mixture of human IL31 (tagged with His) (30 μg) and purified human IL31-IgG (200 μg). Blood was drawn from the retro-orbital sinus at the indicated time points. Plasma was extracted and the presence of IL31 and IL31-IgG proteins was detected by Western blot using anti-His HRP conjugated antibodies. Mouse-IgG light chain served as a loading control. (**D**) U87 human glioblastoma cells were transfected with a STAT3 luciferase reporter plasmid. After 16 hours in serum-free medium, cells were cultured in the presence of rhIL31, IL31-IgG, or SP-IgG at the indicated concentrations for 6 hours. Cells were harvested and lysed. Luciferase substrate was added to cell lysates and bioluminescence was quantified. The experiment was performed in triplicate. *$p < 0.05$.

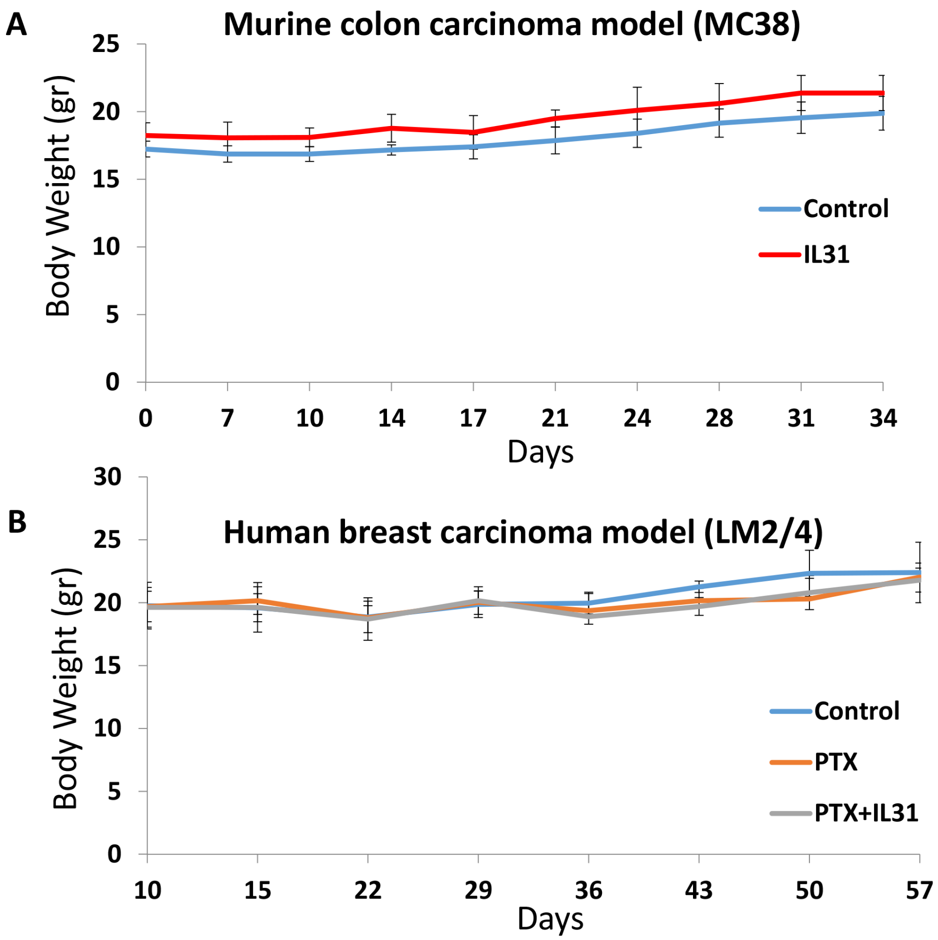

Supplementary Figure 4: Body weight of mice treated with IL31. (**A**) C57/bl mice bearing MC38 (from Figure 3D) or (**B**) NOD-SCID mice bearing LM2-4 (from Figure 7A) were monitored for their body weight during the treatment period. No significant difference was observed in body weight between the different treatment and control arms.
